# Application of Epidemiology Model on Complex Networks in Propagation Dynamics of Airspace Congestion

**DOI:** 10.1371/journal.pone.0157945

**Published:** 2016-06-23

**Authors:** Xiaoxu Dai, Minghua Hu, Wen Tian, Daoyi Xie, Bin Hu

**Affiliations:** National Key Laboratory of Air Traffic Flow Management, Nanjing University of Aeronautics and Astronautics, Nanjing, China; Beihang University, CHINA

## Abstract

This paper presents a propagation dynamics model for congestion propagation in complex networks of airspace. It investigates the application of an epidemiology model to complex networks by comparing the similarities and differences between congestion propagation and epidemic transmission. The model developed satisfies the constraints of actual motion in airspace, based on the epidemiology model. Exploiting the constraint that the evolution of congestion cluster in the airspace is always dynamic and heterogeneous, the SIR epidemiology model (one of the classical models in epidemic spreading) with logistic increase is applied to congestion propagation and shown to be more accurate in predicting the evolution of congestion peak than the model based on probability, which is common to predict the congestion propagation. Results from sample data show that the model not only predicts accurately the value and time of congestion peak, but also describes accurately the characteristics of congestion propagation. Then, a numerical study is performed in which it is demonstrated that the structure of the networks have different effects on congestion propagation in airspace. It is shown that in regions with severe congestion, the adjustment of dissipation rate is more significant than propagation rate in controlling the propagation of congestion.

## Introduction

**Fig 1:**
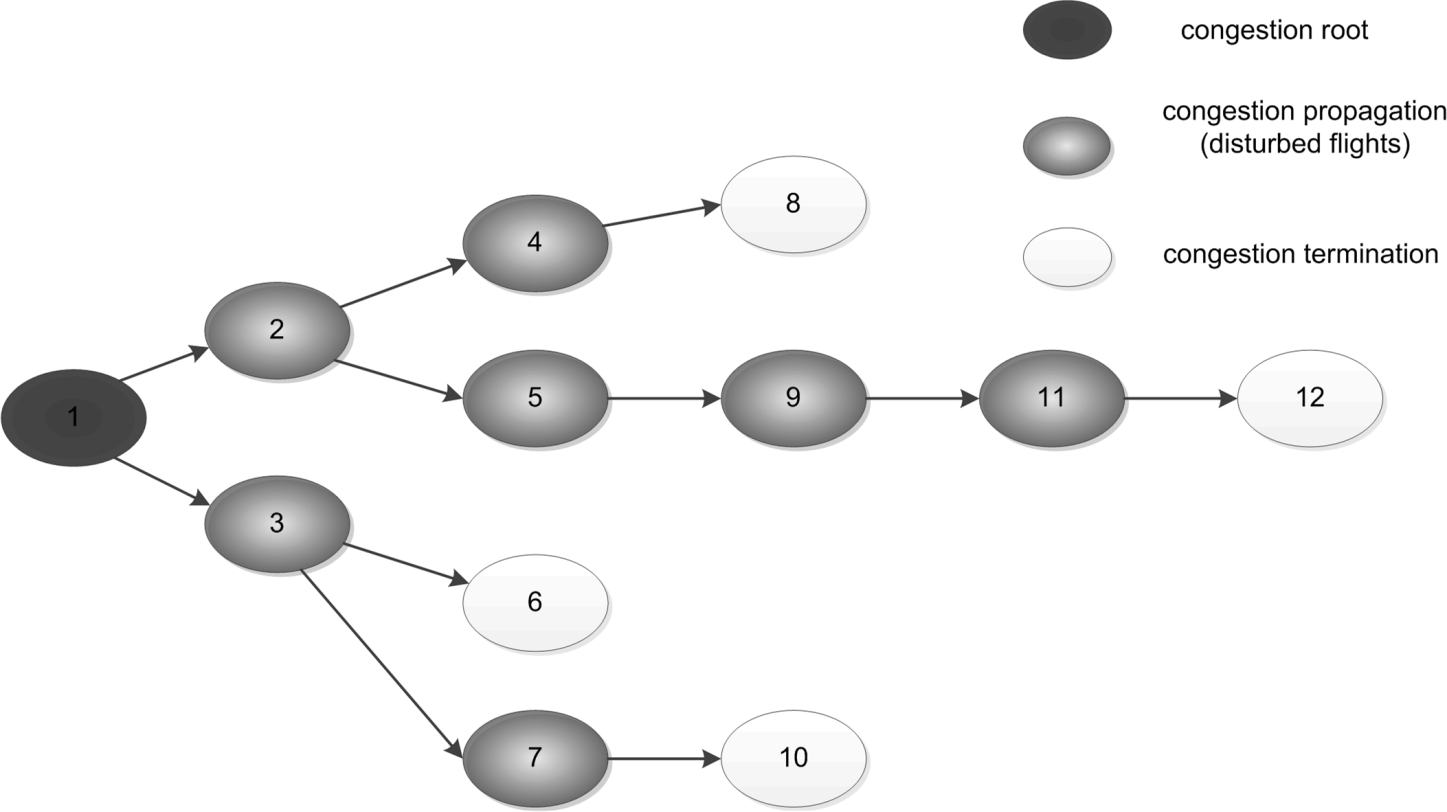
Congestion propagation tree.

Air traffic congestion represents a greater need for airspace capacity. Its propagation in complex air transport networks [1–6] is a paradigmatic example of the way in which a distributed transport system moves towards collapsing. To date most of the research in the propagation of delay or congestion in complex networks in space and time, has focused on road transport. The propagation of congestion in road networks can be analyzed at the macroscopic [7–10], medium [11–13] and microscopic [14–16] levels. However, research in the propagation of congestion or delay in air transport networks is limited, divided into airport [17–21] and airspace [22–24] networks. As shown in Fig 1, the propagation of airspace congestion has traditionally been described as graphs with vertices representing flights and edges representing connectivity. These graphs are referred to as propagation trees [25], and usually use delay or flow distribution for representation. Although simplistic, the propagation tree is useful in understanding how delay or congestion propagates directly through an air transport network. Because of the complexity of air transport networks [26], evolution of congestion within them possesses the characteristics of propagation in complex networks. Delay propagation occurs when late arrivals at an airport cause late departures, identifying the cause but not the mechanism of propagation. The basic regulation and trends of propagation dynamics can be obtained from observation of flow distribution. It should be noted also that congestion propagation in airspace is different from that in road networks. This is because it proceeds in ‘n’ dimensions due to the different altitudes of the same routes in which flights operate on schedules.

For reasons above, this paper pays particular attention to modeling and simulation which have been shown to provide a valuable research tool for the exploration of fundamental laws and trends of congestion propagation in airspace. This research introduces a modeling framework to analyze and visualize the propagation dynamics in air transport networks. In particular, it is notable that many models or data on congestion can be applied successfully to other domains, for example, the spread of infectious diseases at a global scale that occurs when infected persons travel across the network, and the modeling and forecasting of epidemic transmission using air traffic data [27–28]. Therefore, it is intuitive to investigate the application of an epidemic model to the propagation of airspace congestion. This paper models congestion propagation by applying an epidemiology model, informed by the results of the analysis of similarities and differences between disease transmission and congestion propagation. The paper attributes congestion to the cross structure on aviation network, but the model could be extended to solve the congestion caused by other reasons, such as bad weather, mechanical failure or airspace restriction.

## Model

**Fig 2:**
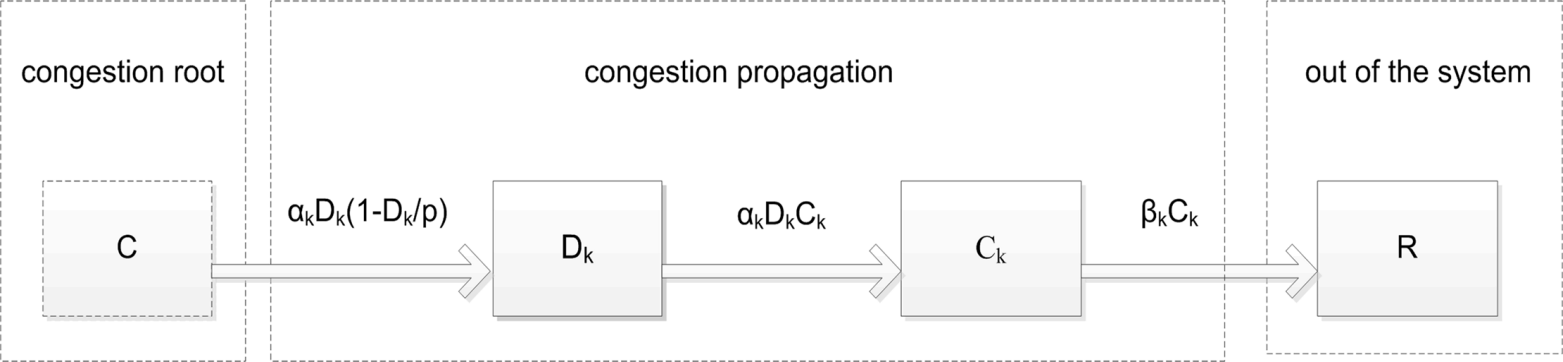
Congestion propagation process.

As seen in Fig 1, due to the branching connectivity between flights, congestion in one flight tends to propagate rapidly down-line to the others in a cascade-like effect. The spread of an epidemic through transmission to individuals in the neighborhood is similar to congestion propagation. Furthermore, flights can be divided into several classes based on the status of congestion in the propagation model, namely congestion roots (C), disturbed flights (D), and removed ones (R) respectively. The disturbed flights are those which are assumed to break the discrete state. Through the analysis of propagation trees, the evolution of disturbed flights results in an index of dynamic propagation. At the micro level, the formation of flights-following or queue is the determinant of congestion, while at the macroscopic level, congestion manifests in flow exceeding capacity. As the congestion characteristics cannot be transmitted to the disturbed flights in seconds, they are not belong to the category of congestion root. At the same time, flights that have either completed their journey or exited specified areas, and therefore, break the connectivity of congestion propagation, are called removed ones, as seen in Fig 2.

One of the epidemiology models, SIR model [29–33], has similar categories to the congestion propagation model: susceptible (S), infected (I), and removed (R). It should be noted that the propagation of diseases is irreversible, in other words, the epidemiology spread is a directional diffuseness, in the same way as congestion propagation. Being different from the classical SIR model, D in the congestion propagation model (1) is a dynamic variable, which increases with congestion propagation, but is limited by the capacity of airspace. And the propagation of airspace congestion is a link emanating from a congested flight to *k* disturbed ones, defining the node degree as *k*, and ending with the removed ones. Let *α*_*k*_ be the propagation rate and *β*_*k*_ be the average percentage of congested flights transiting to the removed ones referred to as the dissipation rate. Therefore, the model is formulated as:

$${\{\begin{array}{l} \frac{dD_{k}}{dt}=\;\text{-}\;\alpha_{k}D_{k}C_{k}\\ \frac{dC_{k}}{dt}=\alpha_{k}D_{k}C_{k}\;\text{-}\;\beta_{k}C_{k} \end{array}}_{}$$ (1)

Taking the logistic increase into consideration, *p* can be denoted as the limited capacity of a certain airspace. As Seen in Fig 2 and expression (2), *D*_*k*_(*t*) and *C*_*k*_(*t*) denote the quantity of the disturbed and congested flights with degree *k* in time *t*, respectively. Thus, the propagation rates *α*_*k*_ and dissipation rates *β*_*k*_ in heterogeneous network may change with congestion propagation or systemic evolution. Moreover, a small variation in *α*_*k*_ and *β*_*k*_ must impact the propagation behaviors in turn.

$${\{\begin{array}{l} \frac{dD_{k}}{dt}=\alpha_{k}D_{k}(1\;\text{-}\;D_{k}/p)\;\text{-}\;\alpha_{k}D_{k}C_{k}\\ \frac{dC_{k}}{dt}=\alpha_{k}D_{k}C_{k}\;\text{-}\;\beta_{k}C_{k} \end{array}}_{}$$ (2)

To simplify the model, we split the heterogeneous network into homogenous ones. As the elementary cell of the airlines, cross traffic can be assumed as a research object, where the homogenous propagation and dissipation rates are constant (denoted as *α* and *β* respectively), as seen in expression (3).

$${\{\begin{array}{l} \frac{dD}{dt}=\alpha D(1\;\text{-}\;D/p)\;\text{-}\;\alpha DC\\ \frac{dC}{dt}=\alpha DC\;\text{-}\;\beta C \end{array}}_{}$$ (3)

**Fig 3:**
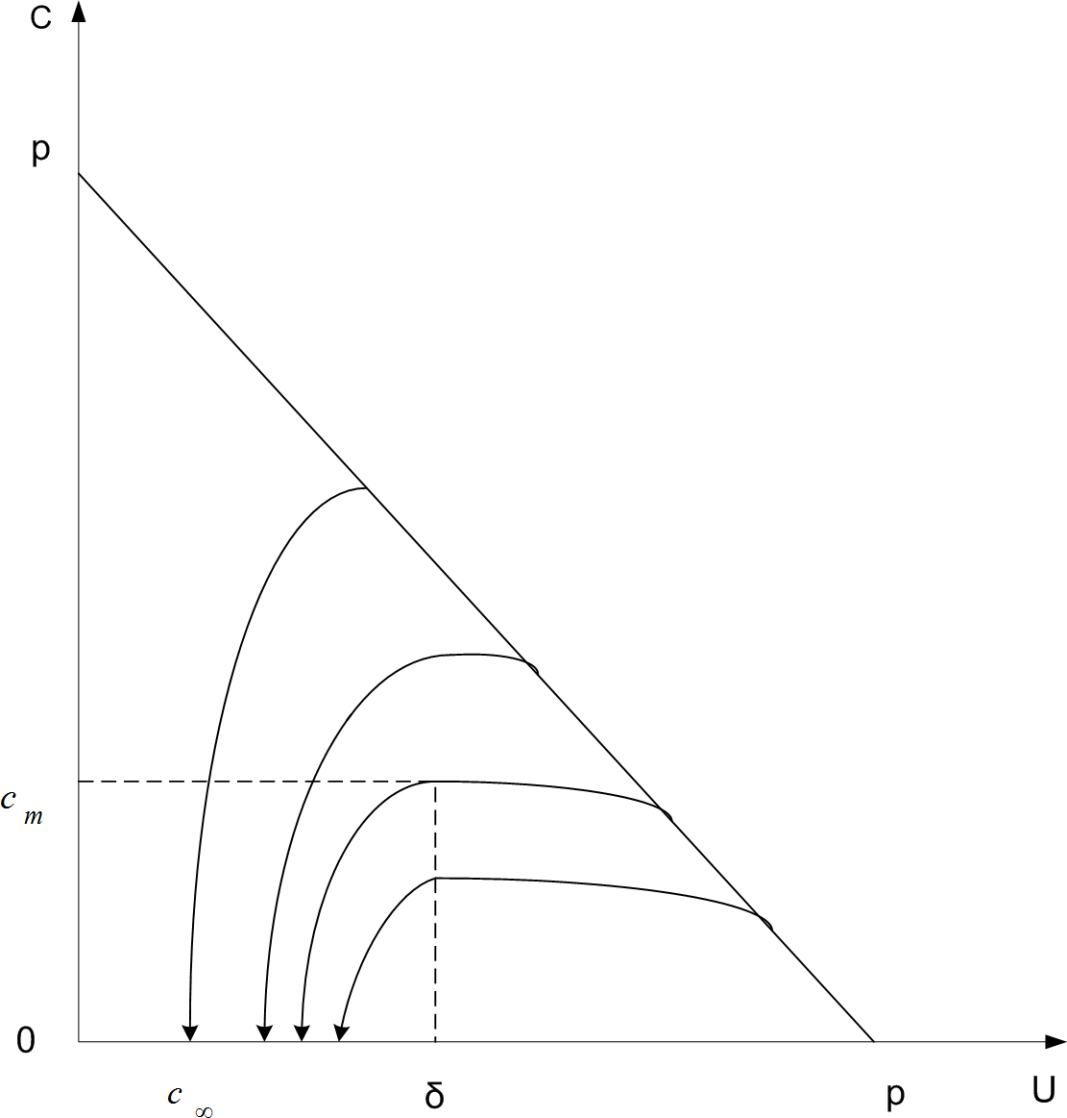
Analysis of phase trajectories.

By analyzing expression (3), we can get the threshold value and balance point of the model. Fig 3 demonstrates the functionary relationship between the congestion flights and disturbed ones, based on expression (4)

C+D+R≤p (4)

1. If *αD* > *β*, *C* would increase and the congestion is subject to exacerbation;
2. If *αD* < *β*, *C* would decrease and the congestion situation mitigates;
3. If *αD* = *β*, *C* would take the local maximum *C*_*m*_.

Based on the above analysis, we get the threshold value *δ* = *β* / *α*. At the same time, Fig 3 shows us *C*_∞_ = 0, which means that the congestion will scatter and disappear without other disturbances. By analyzing expression (3), we can also get the balance point of congestion, as seen in expression (5).

$$\left(C^{*},D^{*})=(1-\beta /\alpha p,\beta /\alpha \right)$$ (5)

One point should be noted that for the convenience purpose, the model of congestion propagation in this paper does not take multiple congestion roots into account. And it is difficult to separate the roots from the congestion data.

### Data analysis and comparison with model prediction

Congestion structure as research object: A) an Approach point (GYA) of ZGGG in China and B) a simulated crossroad in airspace. Evolution of the largest congestion cluster on the timeline: C) May 19, 2014 on GYA and D) simulation results.

**Fig 4:**
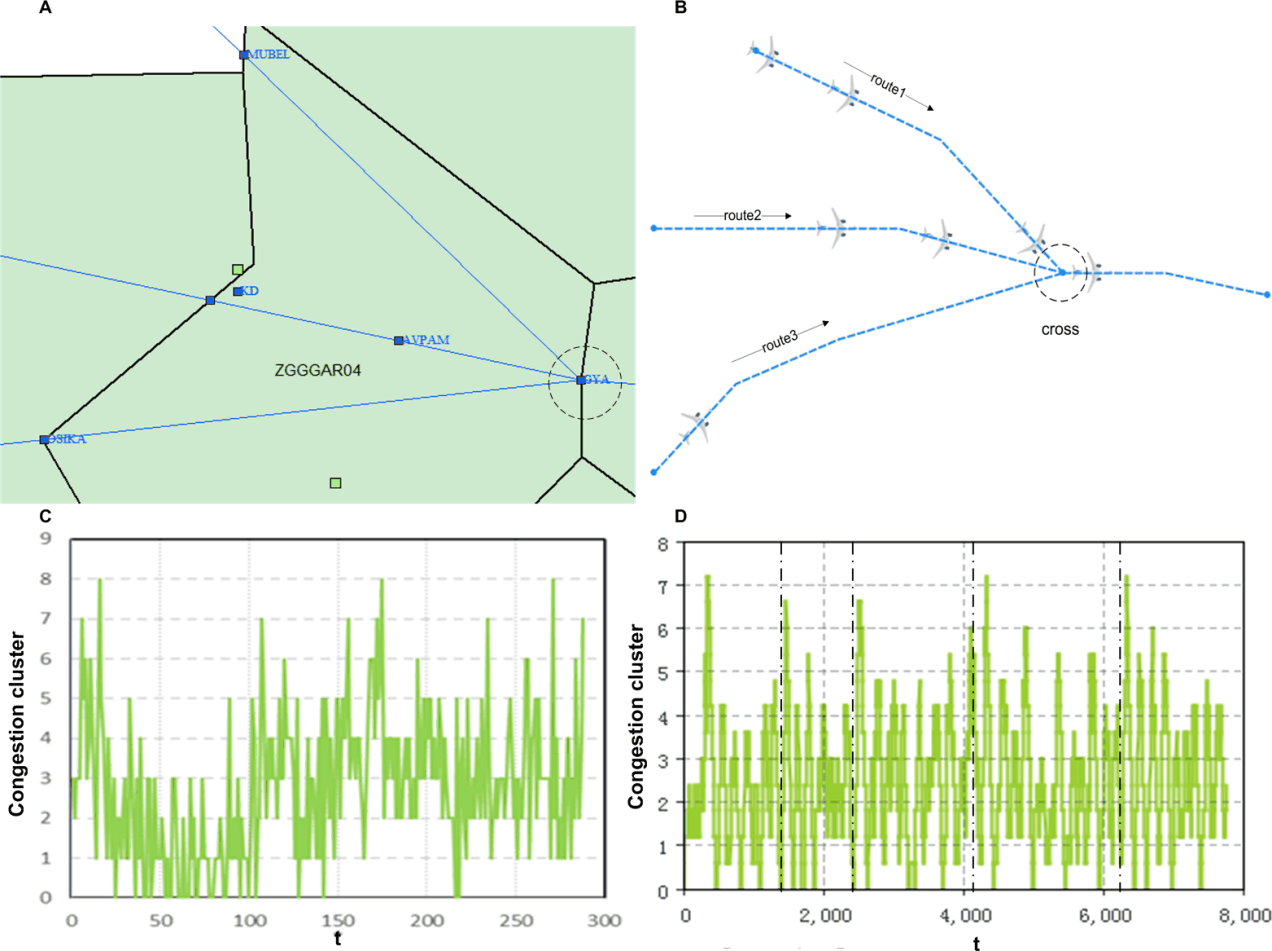
Comparison reality with simulation.

The Guangzhou (ZGGG) Approach area is one of the busiest airspaces in China, with the second highest throughput of 394,403 departure and arrival flights in 2013. The approach point GYA is one of the major ones in the ZGGG Approach area, merging three in-routes and one out-route. The network is one-way, directing flights into ZGGG. Fig 4A shows the structure of the network using GYA as the connecting point. Analysis of the arrival of the in-routes shows that they approximate a Poisson distribution, with the mean values 2.9, 3.0 and 1.2, and corresponding standard deviations of 2.0, 2.0 and 1.2 respectively.

As can be seen in Fig 4C, flow exceeded capacity on 19 May 2014 when the airspace was congested due to bad weather. Studying the structure of GYA, we simulate a similar circumstance in which the in-degree is 3 and out-degree is 1 (see Fig 4B) to observe the propagation of congestion. In the simulation, the length of incident edges is set to the same values as in [34] of 10000m. The flights on the incident edges travel at 100m/s, the interval is 10 minutes, while at the same time, the arrivals obey an index distribution for 30 minutes interval, and the capacity is assumed to be 12 flights per hour. Taking another look at it, the junction in the airspace can be deemed as a service counter of a queuing system, which serves a flight once, with a service time of 10 minutes. The queue before the intersection is the congestion cluster, and the evolution of congestion in the simulated scenario can be seen in Fig 4D. Comparing Fig 4C and Fig 4D, the similarities in periodicity, oscillation and damping can be seen. At the same time, there are differences due to the schedule on in-routes.

**Fig 5:**
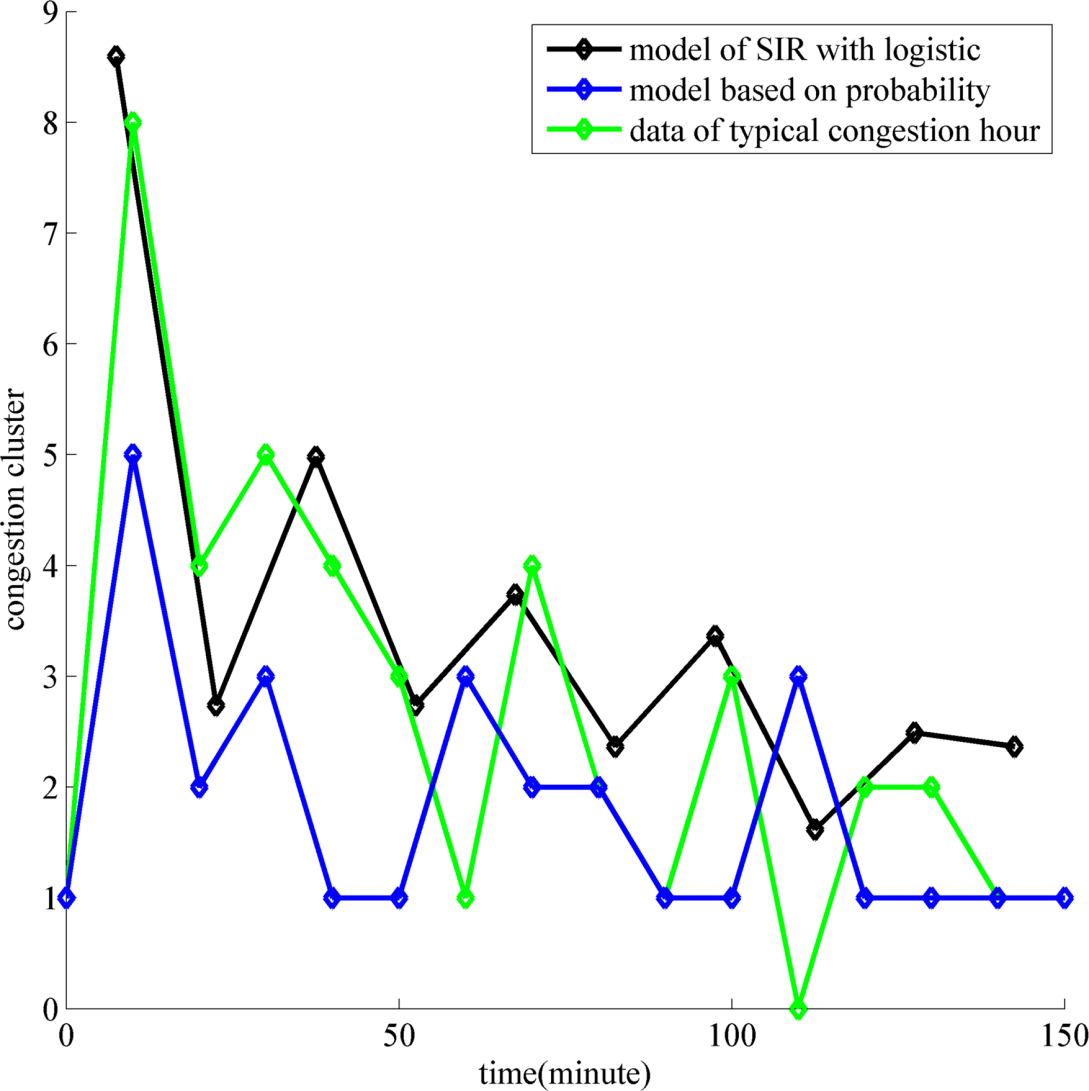
Comparison model with reality and model with model.

To gauge forecast accuracy, we introduce the model based on the equal probability, which is common to flow forecast [35]. Intercepting the congestion period on the typical day, we can find the real data displaying non-uniform attenuation due to the random event. The model of congestion propagation takes on attenuation uniformity ignoring the other disturbances. To aid comparisons with the discrete historical data, we process the computed results of SIR with logistic. Multiply the expectation of the model by its frequency to get the new discrete model as seen in Fig 5. Research on the evolution of peak congestion value in the amplitude and phase difference is more important in the congestion propagation. The congestion propagation may have *n* peaks. *A*_*si*_, *A*_*hi*_ and *A*_*pi*_ are the *ith* peak amplitude value of SIR with logistic, historical data and probability model, respectively. At the same time, *η*_*si*_, *η*_*hi*_ and *η*_*pi*_ are the *ith* peak phase of SIR with logistic, historical data and probability model, respectively. Based on the above premises, the following expression (6) and expression (7) are applied to compare the two models, namely the model of SIR with logistic and the model based on probability.

$$\Delta A_{s}=\frac{1}{n}\sum_{i=1}^{n}\left|A_{si}-A_{hi}\right|,\;\Delta A_{p}=\frac{1}{n}\sum_{i=1}^{n}\left|A_{pi}-A_{hi}\right|$$ (6)

$$\Delta \eta_{s}=\frac{1}{n}\sum_{i=1}^{n}\left|\eta_{si}-\eta_{hi}\right|,\;\Delta \eta_{p}=\frac{1}{n}\sum_{i=1}^{n}\left|\eta_{pi}-\eta_{hi}\right|$$ (7)

Based on the expression (6), expression (7) and Table 1, we get the results that Δ*A*_*s*_ < Δ*A*_*p*_ and Δ*η*_*s*_ < Δ*η*_*p*_. We obtain the conclusion that the model of congestion propagation (the model of SIR with logistic) is more accurate than the model based on probability in describing the value and time of the congestion peak.

**Table 1:** Amplitude and phase difference of two models compared with historical data.

	Amplitude Difference		Phase Difference	
peak	Model of SIR with logistic *vs*. Historical data	Model based on probability *vs*. Historical data	Model of SIR with logistic *vs*. Historical data	Model based on probability *vs*. Historical data
1	0.5	3	3	0
2	0	2	7	0
3	0.2	1	2	10
4	0.3	1	2	20
5	1.5	1	2	20

Assuming that the air transport networks are homogenous, the mechanism and trend of congestion propagation in airspace can be described by expression (3). Although, the real-world networks are heterogeneous, in which the nodes degree and edge length have different values, damping and oscillation trends of congestion propagation are same as homogenous networks. Research on the propagation dynamics model on homogenous networks is useful in understanding flow management particularly congestion and dissipation trends.

### Numerical Analysis

The research on how the main parameters affect the process of propagation is necessary to get define fundamental law of congestion propagation, when it can be regarded as an open loop system.

**Fig 6:**
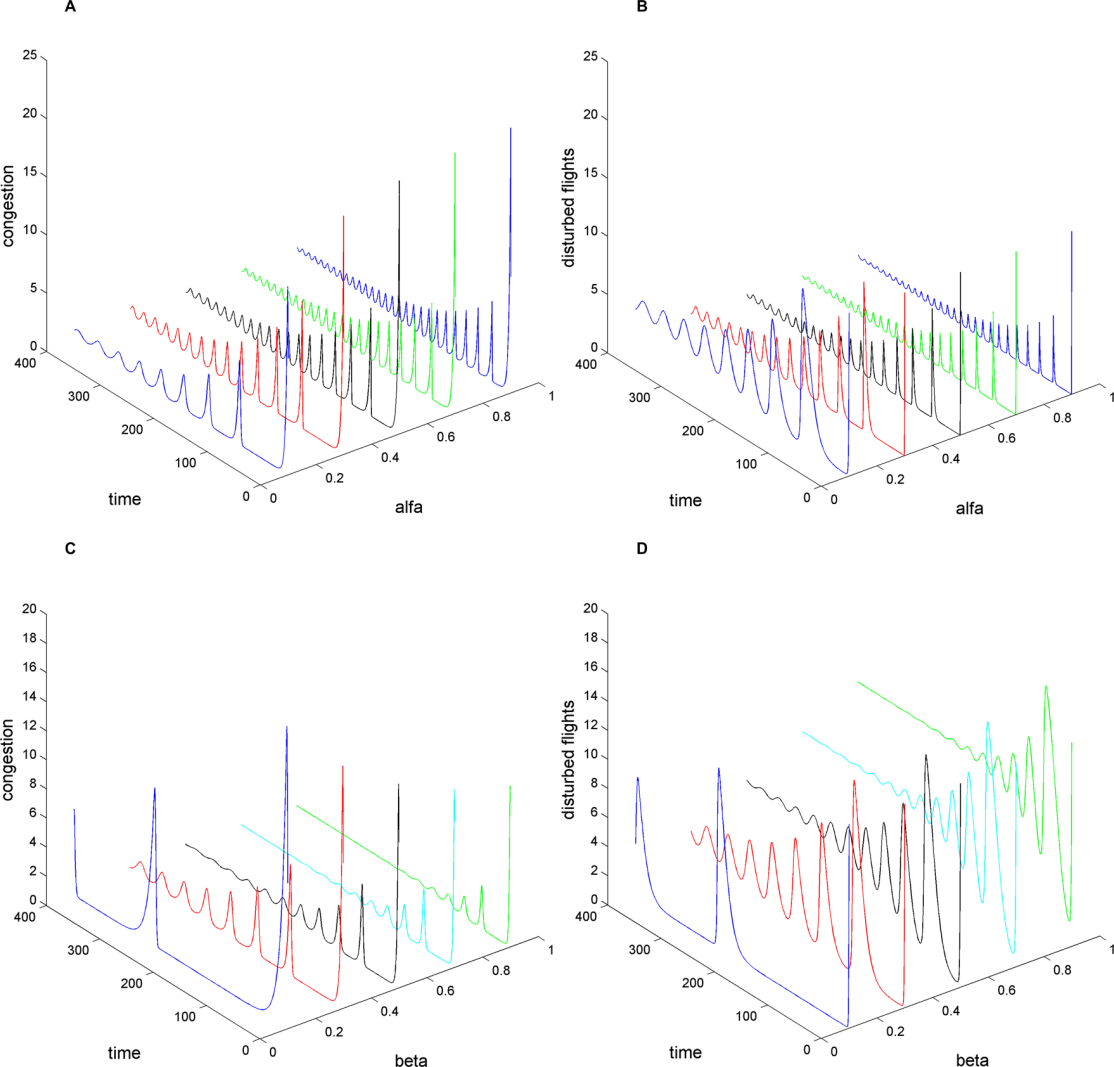
Effect of the main parameters in the evolution of congestion and disturbed flights. The largest congestion cluster per unit time: A) for the parameter alfa setting *β* = 1/3 and C) for the parameter beta setting *α* = 0.1. The disturbed flights per unit time: B) for the parameter alfa setting *β* = 1/3 and D) for the parameter beta setting *α* = 0.1.

**Fig 7:**
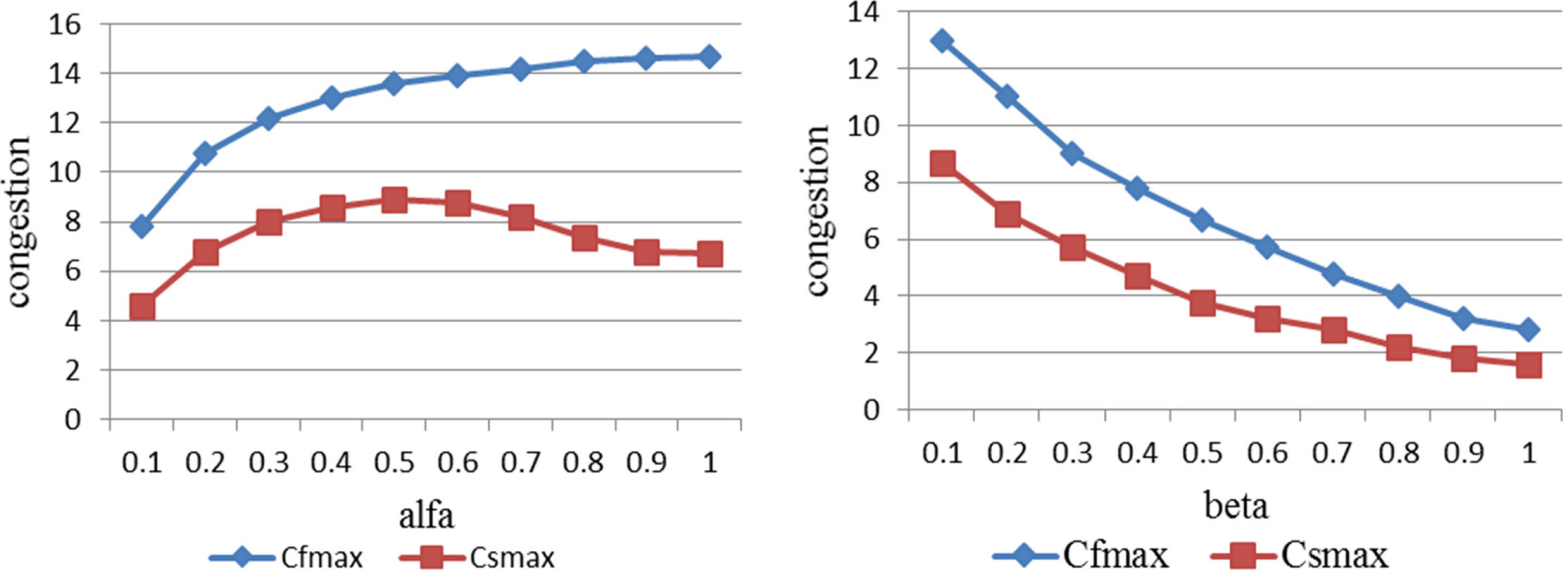
Congestion peaks with main parameters. (A) Congestion peaks with *α* values (0.1–1). (B) Congestion peaks with *β* values (0.1–1).

Fig 6A and Fig 6B show the evolution of congestion and disturbed flights with propagation rate (*α*). Obviously, *α* is a key factor in the prediction of the congestion peak values. Research on congestion propagation should be focused on the maximum value and the duration of congestion. Since propagation presents the trend of oscillation and damping, the first peak value is the maximum value in the propagation. For controllers, they need to predict the congestion peak in time and alleviate traffic congestion before exacerbation. Based on the requirement, we place emphasis on the relationship between peak values and *α* values. Fig 7A shows the peak value (*C*_*f* max_) and sub-peak value (*C*_*s* max_) change with *α* values, in which *C*_*f* max_ increases monotonically and the slope decreases with *α* (*α* = 0.1 ∼ 1). Meanwhile, *C*_*s* max_ with *α* becomes non-monotonic, and *C*_*s* max_ gets the maximum value where *α* = 0.5. At the same time, the values of *C*_*s* max_ have small variations where *α* = 0.4 ∼ 0.6 and *α* = 0.9 ∼ 1. For controllers, a small adjustment of the propagation ratio cannot achieve the expectation to relieve the congestion pressure in very crowded airspaces. Fig 6C and Fig 6D show the evolution of congestion and disturbed flights under different *β* values. Through observation of peak values (*C*_*f* max_) and sub-peak values (*C*_*s* max_) shown in Fig 7B, it can be seen that *C*_*f* max_ and *C*_*s* max_ decrease monotonically with *β* (*β* = 0.1 ∼ 1). By comparison, *β* is more sensitive than *α* to the peak of congestion. Based on the conclusion, the dissipation rate of congestion is more significant in controlling congestion than the propagation rate.

### Conclusion

This paper has modeled the congestion propagation to predict the evolution of flow in crowded airspace, applying the epidemiology models (SIR and SIR with logistic). By comparison and analysis, SIR with logistic has been shown to accurately describe the evolution of congestion peak in amplitude and phase difference. In the numerical analysis, it has been shown that two parameters of propagation rate and dissipation rate play key roles in congestion propagation, especially in the determination of the peak value. In severely congested airspaces, the dissipation rate is more effective in controlling congestion cluster.

Although the size of the largest congested cluster displays in the data a high variability from one hour to the next, the universal law of congestion propagation is invariable. The numerical study has shown the effect of the main parameters on the model and their relevance to the evolution of congestion in the complex networks. Furthermore, the proposed model offers the possibility of evaluating the effects of interventions in the system before implementation.

## Discussion

This paper goes beyond our previous paper [36] in modeling congestion propagation in air traffic management, by applying SIR, which is verified by sample airports. This paper distinguishes the airspace congestion propagation from airport delay propagation, and improves SIR to SIR with logistic which reflects the propagation characteristics in airspace sufficiently. This work is being extended to other traffic situations due, for example, bad weather, mechanical failure or airspace restriction.

## Supporting Information

S1 Dataset flow distribution of in-routes in GYA. (XLS)

S1 Results the result of the simulation in calculating parameter *α*. (TIF)

S2 Results the result of real operation in calculating parameter *α* on GYA. (XLS)
